# Oxygen—Its Agency in Therapeutics

**Published:** 1887-03

**Authors:** B. M. Lawrence


### OXYGEN, ITS AGENCY IN THERAPEUTICS.

BY B. M. LAWRENCE, M. D.

No. 4.

In order to cure any form of disease, no matter what method of treatment is employed, the laws and conditions of health must not be overlooked or neglected, this is especially true respecting the oxygen inhalation system. But, before calling attention to the subject of hygiene, we must not fail to mention one or two more of the many eminent men who may justly be regarded as pioneers in this and other branches of medical reform. Prof. J. R. Buchanan—who has an office in his residence for dispensing the oxygen treatment—recently contributed to one of the leading eclectic journals a series of articles on oxygen and ozone for the cure of disease in which he refers to the remarkable claims and discoveries of Dr. U. F. Mayo, with whom the writer, some time since, conducted a series of experiments with oxygen compounds, which resulted in the organization of a company provided with a chemical laboratory, and all the apparatus required for manufacturing, medicating and compressing into wrought iron cylinders, pure portable compound oxygen, ready for the use of patients or physicians. By this method the growing demand for a home treatment of real gas can be supplied differing entirely from the peroxide of hydrogen and various other preparations which are found to contain no free oxygen, and are therefore of but little real value.

Dr. H. Hutchins has recently made some most remarkable cures with this treatment. He is the author of a small work on "Oyxgen, Nature's Universal Remedy," from which, if space would permit, we could quote at length. He says, "The human body is a self-mending machine, needing, when ill, only the material of repairs, and then to be let alone—an important fact to be remembered." He regards proper attention to diet or pure food, with perfect digestion and assimilation, as most essential adjuncts to the inhalation of oxygen and its compounds. He fully agrees with all scientists who claim that oxygen is, in many respects, the most important element in nature; that it is indispensible to every form of life, in fact, that life becomes extinct if the supply is cut off for only a moment, that all the changes which are continually transpiring in the body are due to its operations.

There can be no health, no enjoyment of existence, no proper performance of bodily functions except oxygen is present in abundance to enliven every portion of the organism. The part which *nitrogen* plays in the human system is one of great importance, and it may be regarded as a vehicle, since it serves to carry the oxygen of the air we breathe into the lungs and to convey the carbonic acid gas and other impurities out of the system at each expiration. It forms a large part of many plants, vegetables and foods, four-fifths of common air, and one-fifth of the flesh of animals, but it is incapable of supporting either combustion or life. The combination of these two gases with ozone forms an *artificial atmospheric air*, which has been, perhaps, inappropriately termed COMPOUND OXYGEN. This mechanical mixture of gases, medicated by passing it through a formula of standard tonics and tinctures, is in every sense a scientific and rational remedy, differing essentially from all other forms of medical inhalations, and may be appropriately termed *the breath af life*, being nothing more or less than the air we breathe, properly medicated with the *oxygen or only life-giving element greatly increased* and all injurious substances excluded, thus rendering it a perfectly natural curative for almost every form of disease. Breathing the excess of oxygen burns up the surplus carbon in the system, which is the great cause of *catarrh*, the forerunner of consumption, and other dangerous diseases. Its inhalation is calculated to clean out and enlarge the capacity of the air cells, and by testing the lungs of a large number of patients, we have never met a case where the *spiometer* did not prove that using the gas increased the ventilating powers of the organs of respiration, and enabled the system to absorb a much greater quantity of oxygen from the atmosphere at each inspiration, while, at the same time, more of the carbonic acid is continually being expelled. In this way the venous blood is arterialized and changed from a dark color to a bright red, which indicates the condition of health and vigor, and the absence of blood impurities, which are the primal cause of all forms of disease. The blood holds in solution all the material out of which the body is made and with which it is daily being repaired. This is equally true of the two great systems of nerves, the *cerebro-spinal* and the *sympathetic* or *ganglionic*. The chemical changes in the blood caused by this great vitalizing agent generate the *vis veta* or vital fluid, which charges the brain, spinal cord, and the entire nervous system like a great electrical or galvanic battery, from which eminate all the life forces which constitute the true healing principle, nature's universal panacea. This oxygen compound, therefore, invigorates the mind and body, the nerves and brain, without exhaustion or subsequent depression. Chemically it generates a greater supply of that vitalized magnetism which is an element essential to perfect health—a most desirable result never obtained by drug medication.

This method of treatment admits of the most direct application—through the blood—conveyed along the arteries and veins to the diseased parts or organs, in all parts of the system, without taxing the stomach, which is almost always already over worked and in need of rest, no matter what the name or nature of the disease, and, as a result, relief may be confidently expected, followed by a permanent cure, if curable, and in the shortest possible space of time.

Finally, in concluding this series of articles, we submit the following reasons why the oxygen treatment should be prescribed by physicians and prefered by patients to other remedial agencies, viz: it assists nature in regulating all the secretions and excretions. It increases, corrects or improves the appetite. It produces increased activity of the digestive, assimilative and excretory functions. It cures chronic constipation. It acts as a universal remedy, and may be used with benefit in all diseases. It sustains the system at all times, and may be safely used in health. It dispenses with all stomach dosing and drugging. It cures, by direct application of the remedy to the disease, wherever located. It controls every symptom more promptly than any other agency. It disposes of poisonous effete matters without taxing the natural functions of the organs. It is perfectly harmless to the most delicate, and never fails to benefit permanently. It imparts new energy to the body and mind, relieving fatigue. It invigorates (rather than stimulates) the nervous system, without producing subsequent depression. It relieves the heart of undue pressure by equalizing the circulation. It increases the powers of respiration, strengthens the chest, and enlarges the capacity of the air cells of the lungs, and, as a result, it purifies the blood, dissolves uric acid, the cause of neuralgia, gout and rheumatism ; burns up surplus carbon, which causes catarrh and consumption. It increases the quantity and improves the quality of "the blood, which is the life."

These conclusions have been reached, after testing the treatment in thousands of cases, and in no instance has it failed to produce satisfactory results when a pure article of gas has been properly administered before the disease had become too deeply seated, providing due attention was paid to the laws and conditions of health and hygiene, some considerations of which will be found in subsequent numbers of HALL'S JOURNAL OF HEALTH.

